# GSK3β Is Increased in Adipose Tissue and Skeletal Muscle from Women with Gestational Diabetes Where It Regulates the Inflammatory Response

**DOI:** 10.1371/journal.pone.0115854

**Published:** 2014-12-26

**Authors:** Martha Lappas

**Affiliations:** 1 Obstetrics, Nutrition and Endocrinology Group, Department of Obstetrics and Gynaecology, University of Melbourne, Victoria, Australia; 2 Mercy Perinatal Research Centre, Mercy Hospital for Women, Heidelberg, Victoria, Australia; Virgen Macarena University Hospital, School of Medicine, University of Seville, Spain

## Abstract

Infection and inflammation, through their ability to increase pro-inflammatory cytokines and chemokines and adhesion molecules, are thought to play a central role in the pathophysiology of insulin resistance and type 2 diabetes. Recent studies have shown that glycogen synthase kinase 3 (GSK3) plays a central role in regulating this inflammation. There are, however, no studies on the role of GSK3 in pregnancies complicated by gestational diabetes mellitus (GDM). Thus, the aims of this study were (i) to determine whether GSK3 is increased in adipose tissue and skeletal muscle from women with GDM; and (ii) to investigate the effect of GSK3 inhibition on inflammation in the presence of inflammation induced by bacterial endotoxin lipopolysaccharide (LPS) or the pro-inflammatory cytokine IL-1β. Human omental adipose tissue and skeletal muscle were obtained from normal glucose tolerant (NGT) women and BMI-matched women with diet-control GDM at the time of Caesarean section. Western blotting was performed to determine GSK3 protein expression. Tissue explants were performed to determine the effect of the GSK3 inhibitor CHIR99021 on markers of inflammation. When compared to women with NGT, omental adipose tissue and skeletal muscle obtained from women with diet-controlled GDM had significantly higher GSK3β activity as evidenced by a decrease in the expression of GSK3β phosphorylated at serine 9. The GSK3 inhibitor CHIR99021 significantly reduced the gene expression and secretion of the pro-inflammatory cytokines TNF-α, IL-1β and IL-6; the pro-inflammatory chemokines IL-8 and MCP-1; and the adhesion molecules ICAM-1 and VCAM-1 in tissues stimulated with LPS or IL-1β. In conclusion, GSK3 activity is increased in GDM adipose tissue and skeletal muscle and regulates infection- and inflammation-induced pro-inflammatory mediators.

## Introduction

The rates of gestational diabetes mellitus (GDM) are increasing worldwide, intensified with advancing maternal age, racial/ethnic disparities, and obesity [1]. While the mother is at high risk of future development of diabetes [2], [3], GDM also conveys significant risks to the children [3], [4].

The usual increase in insulin resistance seen in late pregnancy [5] is enhanced in women with GDM [5]–[7]. The resultant increase in glucose, lipids, and amino acids disrupts the intrauterine milieu; the fetus is exposed to these excessive fuel sources resulting in increased fetal adiposity and/or macrosomia [8], [9] and thus risk for disease postnatally. Pro-inflammatory cytokines are thought to be central mediators of this enhanced peripheral insulin resistance [10], [11]. In support, adipose tissue and skeletal muscle from pregnant women synthesise and secrete a number of inflammatory mediators [12]–[16] that are enhanced in women with GDM [16]–[19] and that have been shown to correlate with fetal adiposity [20]–[22].

Activation of Toll-like receptor (TLR) signalling pathways by bacterial products are also thought to play a role in the pathophysiology of diabetes. For example, the TLR4 ligand bacterial lipopolysaccharide (LPS) from the Gram-negative intestinal microbiota induces features of metabolic diseases such as inflammation and insulin resistance [23]. Interestingly, pregravid obesity is associated with increased maternal endotoxemia [19], and LPS has been shown to induce the expression of pro-inflammatory cytokines in adipose tissue and skeletal muscle from pregnant women [13], [15].

Studies by Martin and colleagues in 2005 first demonstrated the role of glycogen synthase kinase 3 (GSK3) in the regulation of inflammation [24]. Glycogen synthase kinase 3 (GSK3) α and β are serine/threonine protein kinases that are involved in the storage of glucose into glycogen. *In vivo*, increased GSK3 activity is an early event in the development of insulin resistance where glycogen synthesis is impaired in type 2 diabetes [25] and inhibition of GSK3 in Zucker diabetic fatty rats leads to an improvement in both insulin action and glucose uptake [26]. Notably, GSK3α/β inhibition has been shown to suppress inflammation in response to a variety of stimuli such as TNF-α, IL-1β and LPS *in vitro* [24]. As such, GSK3 has been shown to play a role in a number of inflammatory diseases [27], [28].

GSK3 activity is increased in skeletal muscle samples and/or adipose tissue from insulin resistance states [25], [29]. Previous studies from my laboratory have shown that GSK3 mRNA expression is increased in adipose tissue and decreased in skeletal muscle from women with GDM [7]. However, a limitation of these studies was that only gene expression was assessed. Importantly, the inactivation of GSK3 activity is induced by phosphorylation at one of its N-terminal serine (Ser) residues: Ser21 for GSK3α and Ser9 for GSK3β. Thus, the aim of this study was to determine the effect of GDM on GSK3α/β protein expression in omental adipose tissue and skeletal muscle. To determine if GSK3 plays a role in adipose tissue and skeletal muscle inflammation induced by endotoxemia (i.e. LPS) or the pro-inflammatory cytokine IL-1β, the effect of the GSK3 inhibitor CHIR99021 was also examined. CHIR99021 is the most selective inhibitor of GSK3α/β kinase activity reported to date, exhibiting>500-fold selectivity for GSK3 over closely related kinases [30].

## Materials and Methods

### Ethics Statement

Written informed consent was obtained from all participating patients. Ethics approval was obtained from the Mercy Hospital for Women's Research and Ethics Committee. Pregnant women were recruited to the study by a clinical research midwife.

### Tissue collection and preparation

Human omental adipose tissue and skeletal muscle (pyramidalis) was obtained from consenting women who delivered healthy, singleton infants at term (>37 weeks gestation). Indications for Caesarean section were breech presentation and/or previous Caesarean section. Tissues were obtained and processed within 15 min of delivery.

Omental adipose tissue (n = 24 patients) and skeletal muscle (n = 24 patients) was obtained from normal glucose tolerant (NGT) women and BMI-matched women with GDM. The clinical details of the patients are presented in Table 1 for omental adipose tissue and Table 2 for skeletal muscle. Women with any underlying medical conditions such as pre-existing diabetes, asthma, polycystic ovarian syndrome, preeclampsia and macrovascular complications were excluded. Women with GDM were diagnosed according to the criteria of the Australasian Diabetes in Pregnancy Society (ADIPS) by either a fasting venous plasma glucose concentrations of ≥5.5 mmol/l glucose, and/or ≥8.0 mmol/l glucose 2 h after a 75 g oral glucose load at approximately 28 weeks gestation. All women with GDM were managed by diet alone. All pregnant women were screened for GDM, and women participating in the normal group had a negative screen. Tissues were thoroughly washed in ice-cold PBS to remove any blood. Dissected fragments were stored at −80°C until assayed as detailed below.

**Table 1:** Clinical characteristics of the adipose tissue study group.

	NGT (n = 12)	GDM-diet (n = 12)
Maternal age (years)	31.8±1.7	34.9±1.5
Maternal BMI at ∼12 wks (kg/m^2^)	33.3±3.4	30.6±2.1
Maternal BMI at delivery (kg/m^2^)	36.8±2.9	33.1±1.6
Gestational weight gain (kg)	9.4±1.8	6.7±2.5
Gestational age at birth (weeks)	38.6±0.1	38.6±0.2
Fetal birth weight (g)	3761±186	3277±153
Fetal Gender	6 Female; 6 Male	7 Female; 5 Male
Maternal OGTT at ∼28 weeks gestation		
…Fasting plasma OGTT (mmol/l)	4.5±0.1	5.3±0.2* ^§^
…1 hour plasma OGTT (mmol/l)	7.5±0.6	10.3±0.3*
…2 hour plasma OGTT (mmol/l)	5.9±0.4	8.8±0.5*

OGTT, oral glucose tolerance test.

Values represent mean±SEM.

*P<0.05 vs. NGT.

**Table 2:** Clinical characteristics of the skeletal muscle study group.

	NGT (n = 12)	GDM-diet (n = 12)
Maternal age (years)	32.7±1.4	32.3±1.6
Pre-pregnancy BMI (kg/m^2^)	32.6±3.5	33.8±2.6
Maternal BMI at delivery (kg/m^2^)	36.4±2.9	36.4±2.7
Gestational weight gain (kg)	10.3±2.5	6.3±1.4
Gestational age at birth (weeks)	38.7±0.3	38.5±0.2
Fetal birth weight (g)	3606±116	3414±129
Fetal Gender	6 Female; 6 Male	6 Female; 6 Male
Maternal OGTT at ∼28 weeks gestation		
…Fasting plasma OGTT (mmol/l)	4.4±0.1	5.4±0.1*
…1 hour plasma OGTT (mmol/l)	5.6±0.4	11.0±0.3*
…2 hour plasma OGTT (mmol/l)	4.9±0.3	9.4±0.5*

OGTT, oral glucose tolerance test.

Values represent mean ±SEM.

*P<0.05 vs. NGT.

### Tissue explant culture

Tissue explants were performed to determine the effect of the GSK3 inhibitor CHIR99021 on inflammation in pregnant adipose tissue and skeletal muscle. For these studies, adipose tissue and skeletal muscle was obtained from non-obese NGT pregnant women, and tissue explants were performed as previously described [12], [13]. Briefly, adipose tissue and skeletal muscle was finely diced and placed in DMEM at 37°C in a humidified atmosphere of 21% O_2_ and 5% CO_2_ for 1 h. Tissues were blotted dry on sterile filter paper and transferred to 24-well tissue culture plates (100 mg for adipose tissue and 50 mg for skeletal muscle). Dissected adipose tissue and skeletal muscle tissues were incubated in 1 ml DMEM (with 100 U/ml penicillin G and 100 µg/ml streptomycin). Tissues were incubated in the absence or presence of 10 µM CHIR99021 (Selleck Chemicals; Houston, TX, USA) for 60 min before the addition of 10 µg/ml LPS (derived from Escherichia coli 026:B6; Sigma-Aldrich, St. Louis, MO, USA) or 1 ng/ml IL-1β (PeproTech; Rocky Hill, NJ, USA) for 20 h. For adipose tissue, additional explants were performed using another GSK3 inhibitor SB216763 (Selleck Chemicals; Houston, TX, USA). For these studies, adipose tissue was incubated in the absence or presence of 20 µM SB216763 for 60 min before the addition of 10 µg/ml LPS for 20 h. After final incubation, tissue and media were collected separately and stored at −80°C for further analysis as detailed below. The concentration of CHIR99021 was based on past studies [31]. Each treatment was performed on tissues obtained from six patients.

### Western blotting

Tissue lysates and Western blotting were prepared as previously described [12]. Twenty micrograms of protein was separated on polyacrylamide gels (Bio-Rad Laboratories; Gladesville, NSW, Australia) and transferred to PVDF. Protein expression was identified by comparison with the mobility of protein standard. Blots were incubated with rabbit polyclonal phosphorylated (Ser21/9) GSK3α/β (#9331; Cell Signalling, Beverly, MA, USA) or rabbit monoclonal total GSK3β (#9315, Cell Signalling, Beverly, MA, USA) diluted 1/1000 in blocking buffer (3% BSA in TBS with 0.05% Tween-20) for 16 h at 4°C. Membranes were viewed and analysed using the Chemi-Doc system (Bio-Rad Laboratories; Gladesville, NSW, Australia). Semi-quantitative analysis of the relative density of the bands in Western blots was performed using Quantity One 4.2.1 image analysis software (Bio-Rad Laboratories; Gladesville, NSW, Australia). The levels of phosphorylated GSK3α/β were normalised to the levels of total GSK3β and fold change was calculated relative to the NGT group.

### RNA extraction and quantitative RT-PCR (qRT-PCR)

Total RNA was extracted from tissues using TRIsure according to manufacturer's instructions (Bioline, Alexandria, NSW, Australia). RNA concentration and purity were measured using a NanoDrop ND1000 spectrophotometer (Thermo Fisher Scientific; Scoresby, Vic, Australia). RNA was converted to cDNA the Tetro cDNA synthesis kit (Bioline, Alexandria, NSW, Australia) according to the manufacturer's instructions. The cDNA was diluted fifty-fold, and 4 µl of this was used to perform RT-PCR using SensiFAST SYBR (Bioline, Alexandria, NSW, Australia)) and 100 nM of pre-designed and validated QuantiTect primers (Qiagen, Chadstone Centre, Vic, Australia). The RT-PCR was performed using a CFX384 Real-Time PCR detection system (Bio-Rad Laboratories; Gladesville, NSW, Australia). Average gene Ct values were normalised to the average GAPDH Ct values of the same cDNA sample. Of note, there was no effect of experimental treatment on GAPDH gene expression. Fold differences were determined using the comparative Ct method. For the explant studies, there was a large variability in the response to LPS or IL-1β which is normal for tissues derived from different patients. Thus, fold change was calculated relative to LPS or IL-1β, which was set at 1.

### Cytokine immunoassays

The release of MCP-1, TNF-α, IL-6 and IL-8 was performed by sandwich ELISA according to the manufacturer's instructions (Life Technologies; Mulgrave, Vic, Australia). The concentration of IL-1β, sICAM-1 and sVCAM-1 in the media was performed by sandwich ELISA according to the manufacturer's instructions (R&D Systems; Minneapolis, MN, USA). All data were corrected for total protein and expressed as either pg or ng per mg protein. The protein content of tissue homogenates was determined using BCA protein assay (Thermo Fisher Scientific; Scoresby, Vic, Australia), using BSA as a reference standard, as previously described [15]. The calculated interassay and intraassay coefficients of variation (CV) were all less than 10%.

### Statistical analysis

**Figure 1:**
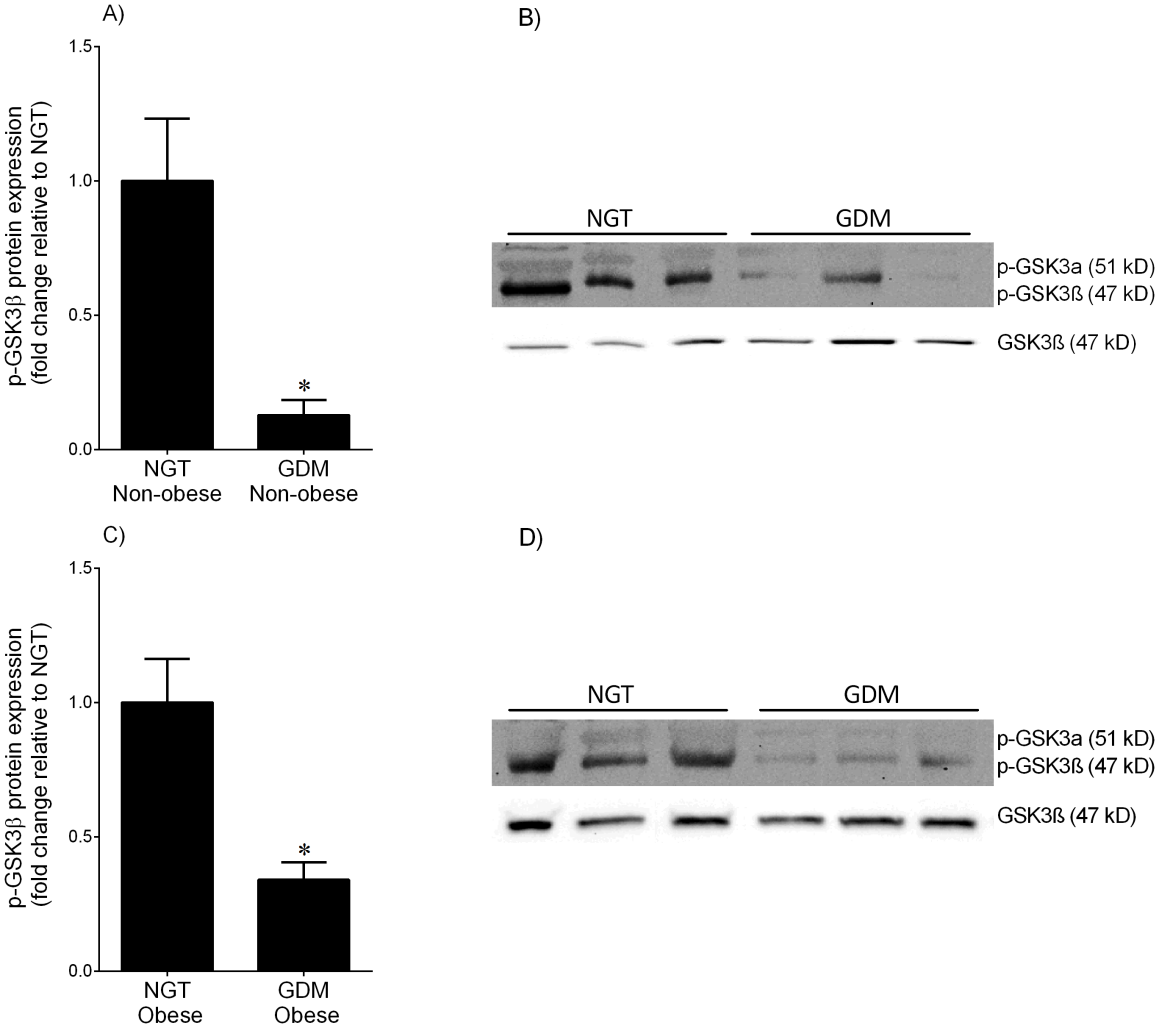
Phosphorylated GSKβ expression in adipose tissue from NGT and GDM women. Omental adipose tissue was obtained from (**A,B**) non-obese and (**C,D**) obese women with NGT (n = 6 patients per group) and diet-controlled GDM (n = 6 patients per group) at the time of term Caesarean section. Phosphorylation of GSK3α at serine 21 (p-GSKα) was very low and thus not analysed further. Phosphorylation of GSK3β at serine 9 (p-GSKβ) was analysed by immunoblotting and normalised to total GSK3β protein expression. The fold change was calculated relative to NGT and data is displayed as mean ±SEM. *P<0.05 vs. NGT (Student's t-test). Representative Western blot from 3 NGT and 3 diet-controlled GDM patients is also shown.

**Figure 2:**
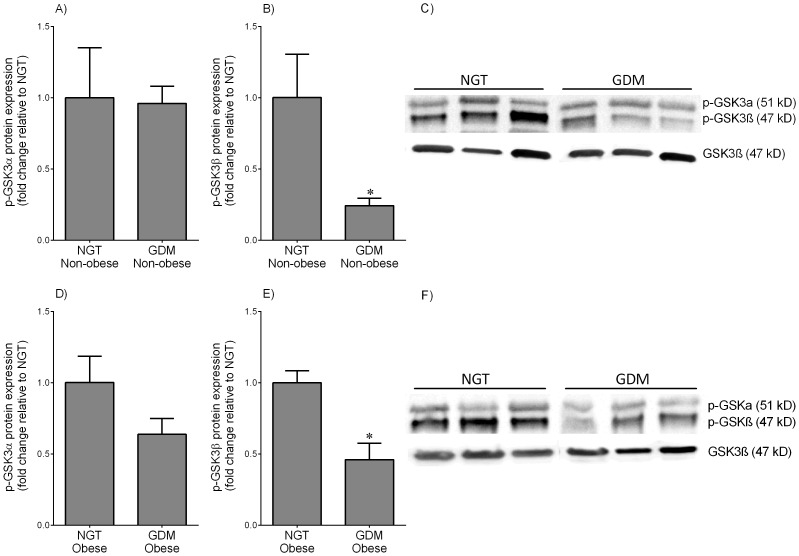
Phosphorylated GSKα/β expression in skeletal muscle from NGT and GDM women. Skeletal muscle was obtained from (**A–C**) non-obese and (**D–F**) obese women with NGT (n = 6 patients per group) and diet-controlled GDM (n = 6 patients per group) at the time of term Caesarean section. Phosphorylation of GSK3α at serine 21 (p-GSKα) and GSK3β at serine 9 (p-GSKβ) was analysed by immunoblotting and normalised to total GSK3β protein expression. The fold change was calculated relative to NGT and data is displayed as mean ±SEM. *P<0.05 vs. NGT (Student's t-test). Representative Western blot from 3 NGT and 3 diet-controlled GDM patients is also shown.

**Figure 3:**
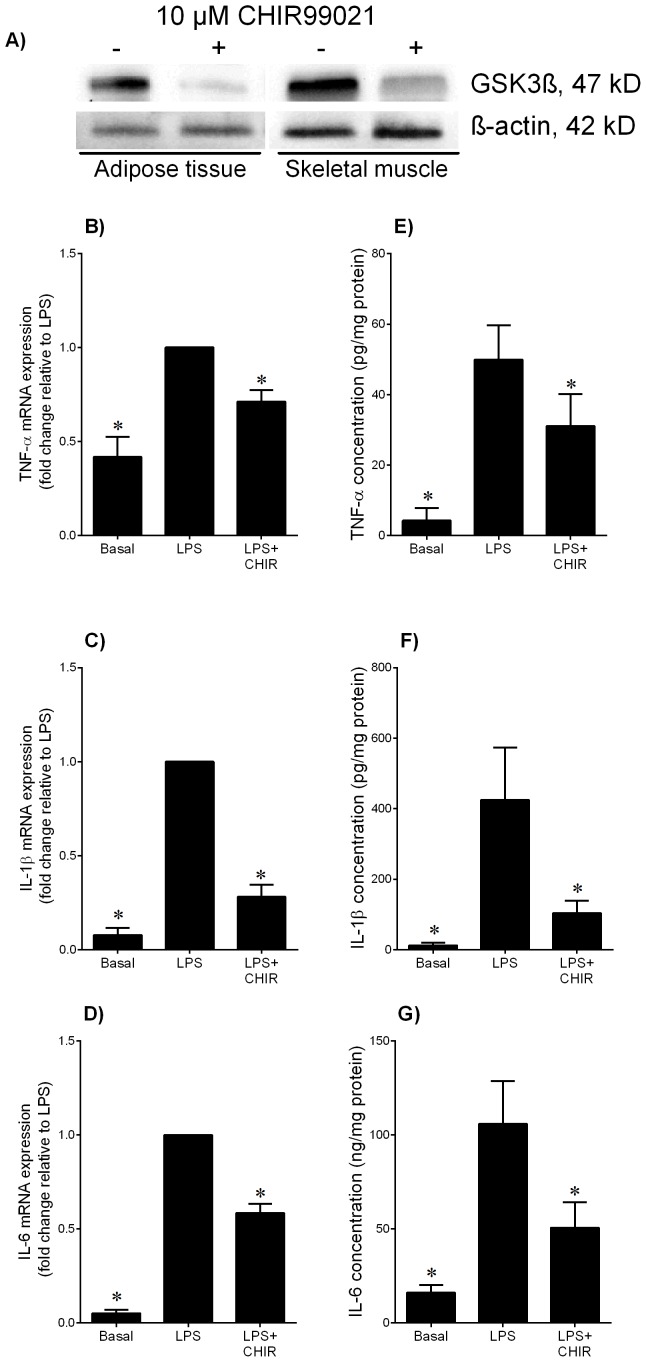
Effect of GSK3 inhibitor CHIR99021 on LPS-induced pro-inflammatory cytokines in adipose tissue. (**A**) Human omental adipose tissue and skeletal muscle were incubated in the absence or presence of 10 µM CHIR99021 (CHIR) for 20 h. Representative Western blot demonstrating GSK3β protein expression. (**B–G**) Human omental adipose tissue was incubated with 10 µg/ml LPS in the absence or presence of 10 µM CHIR99021 (CHIR) for 20 h (n = 6 patients). (**B–D**) Gene expression for TNF-α, IL-1β and IL-6 was analysed by qRT-PCR. Gene expression was normalised to GAPDH mRNA expression and the fold change was calculated relative to LPS. Data displayed as mean ±SEM. *P<0.05 vs. LPS (one-way ANOVA). (**E–G**) The incubation medium was assayed for concentration of TNF-α, IL-1β and IL-6 release by ELISA. Data displayed as mean ±SEM. *P<0.05 vs. LPS (one-way ANOVA).

**Figure 4:**
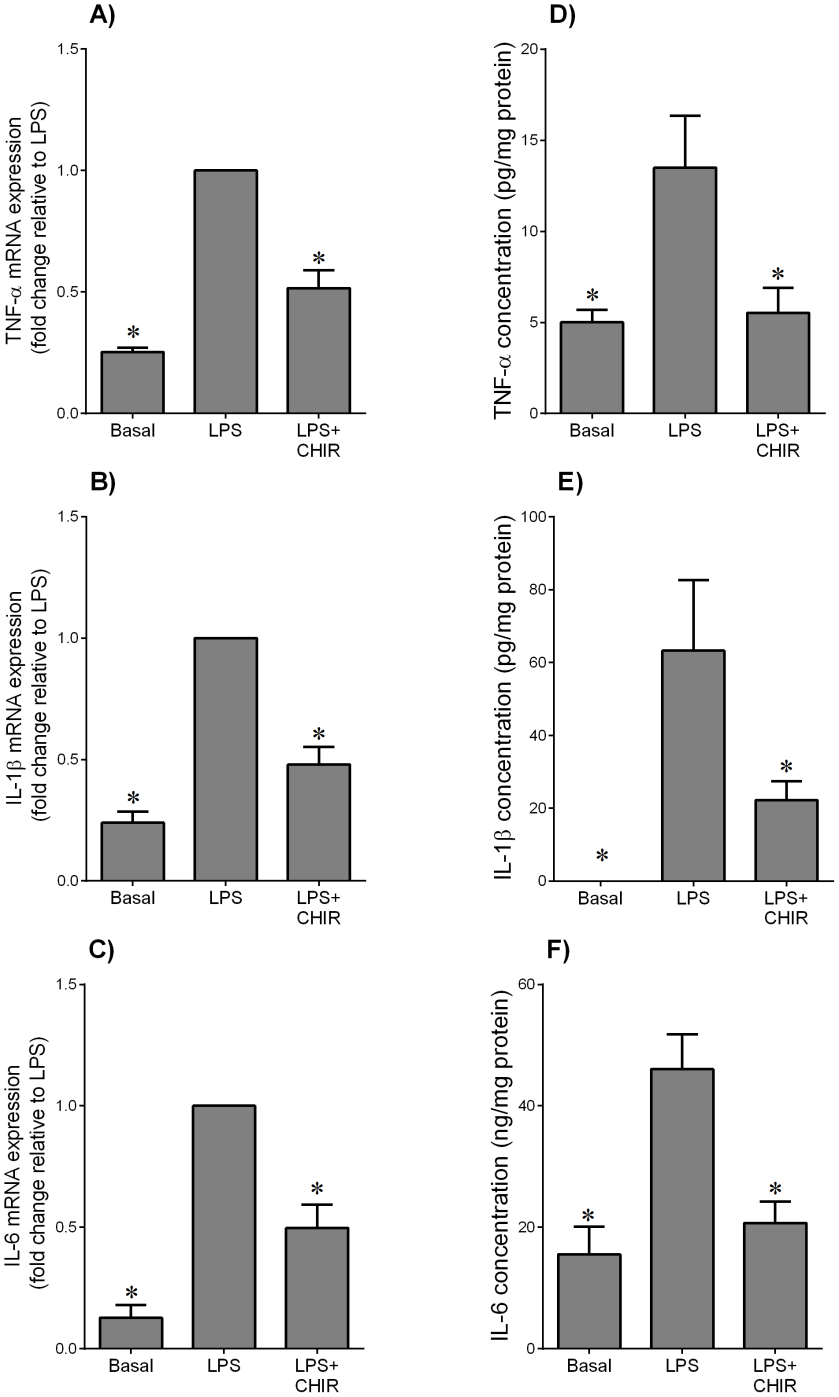
Effect of GSK3 inhibitor CHIR99021 on LPS-induced pro-inflammatory cytokines in skeletal muscle. Human skeletal muscle was incubated with 10 µg/ml LPS in the absence or presence of 10 µM CHIR99021 (CHIR) for 20 h (n = 6 patients). (**A–C**) Gene expression for TNF-α, IL-1β and IL-6 was analysed by qRT-PCR. Gene expression was normalised to GAPDH mRNA expression and the fold change was calculated relative to LPS. Data displayed as mean ±SEM. *P<0.05 vs. LPS (one-way ANOVA). (**D–F**) The incubation medium was assayed for concentration of TNF-α, IL-1β and IL-6 release by ELISA. Data displayed as mean ±SEM. *P<0.05 vs. LPS (one-way ANOVA).

**Figure 5:**
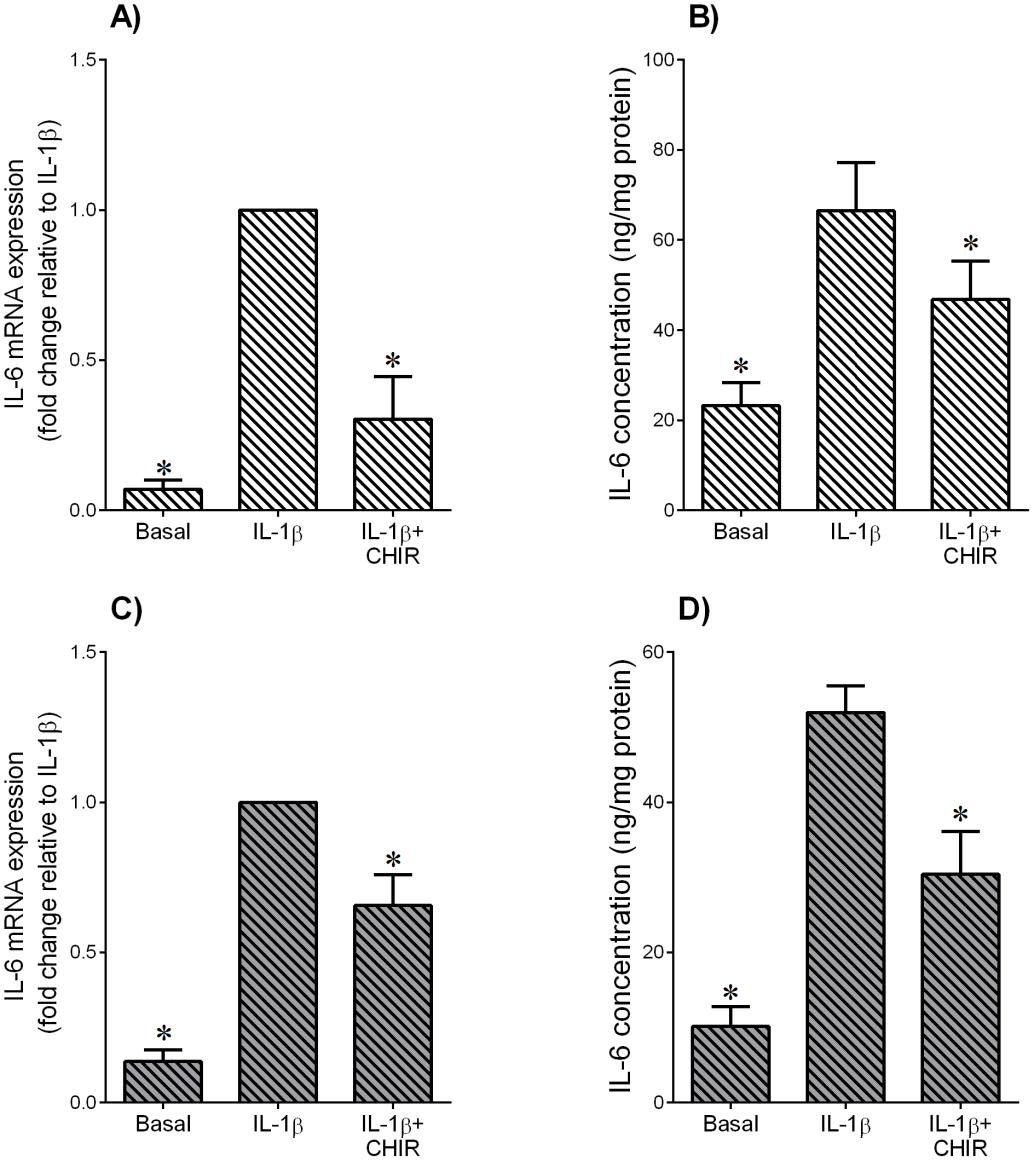
Effect of GSK3 inhibitor CHIR99021 on IL-1β-induced pro-inflammatory cytokine IL-6 in adipose tissue and skeletal muscle. Human (**A,B**) omental adipose tissue and (**C,D**) skeletal muscle were incubated with 1 ng/ml IL-1β in the absence or presence of 10 µM CHIR99021 (CHIR) for 20 h (n = 6 patients). (**A,C**) Gene expression for IL-6 was analysed by qRT-PCR. Gene expression was normalised to GAPDH mRNA expression and the fold change was calculated relative to IL-1β. Data displayed as mean ±SEM. *P<0.05 vs. IL-1β (one-way ANOVA). (**B,D**) The incubation medium was assayed for concentration of IL-6 release by ELISA. Data displayed as mean ±SEM. *P<0.05 vs. IL-1β (one-way ANOVA).

**Figure 6:**
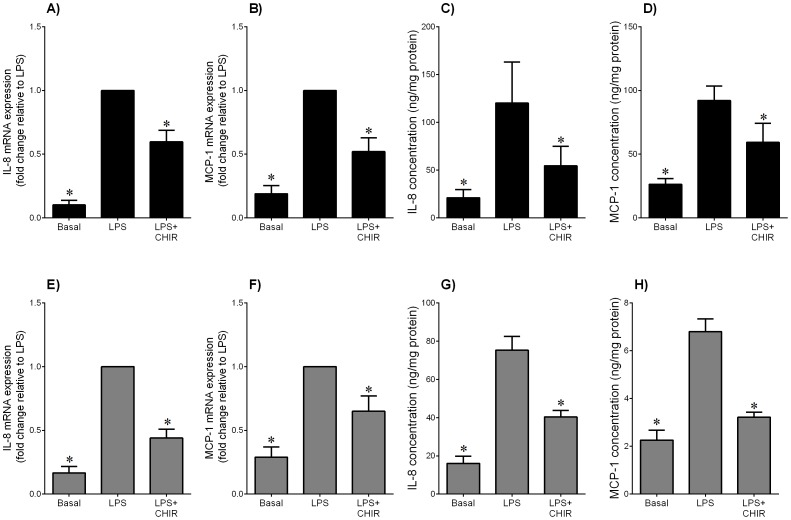
Effect of GSK3 inhibitor CHIR99021 on LPS-induced pro-inflammatory chemokines in adipose tissue and skeletal muscle. Human (**A–D**) omental adipose tissue and (**E–H**) skeletal muscle were incubated with 10 µg/ml LPS in the absence or presence of 10 µM CHIR99021 (CHIR) for 20 h (n = 6 patients). (**A,B,E,F**) Gene expression for IL-8 and MCP-1 was analysed by qRT-PCR. Gene expression was normalised to GAPDH mRNA expression and the fold change was calculated relative to LPS. Data displayed as mean ±SEM. *P<0.05 vs. LPS (one-way ANOVA). (**C,D,G,H**) The incubation medium was assayed for concentration of IL-8 and MCP-1 release by ELISA. Data displayed as mean ±SEM. *P<0.05 vs. LPS (one-way ANOVA).

**Figure 7:**
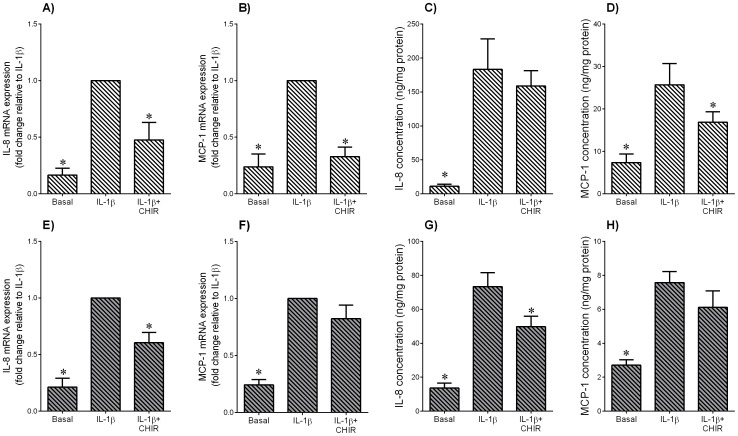
Effect of GSK3 inhibitor CHIR99021 on IL-1β induced pro-inflammatory chemokines in adipose tissue and skeletal muscle. Human (**A–D**) omental adipose tissue and (**E–H**) skeletal muscle were incubated with 1 ng/ml IL-1β in the absence or presence of 10 µM CHIR99021 (CHIR) for 20 h (n = 6 patients). (**A,B,E,F**) Gene expression for IL-8 and MCP-1 was analysed by qRT-PCR. Gene expression was normalised to GAPDH mRNA expression and the fold change was calculated relative to IL-1β. Data displayed as mean ±SEM. *P<0.05 vs. IL-1β (one-way ANOVA). (**C,D,G,H**) The incubation medium was assayed for concentration of IL-8 and MCP-1 release by ELISA. Data displayed as mean ±SEM. *P<0.05 vs. IL-1β (one-way ANOVA).

**Figure 8:**
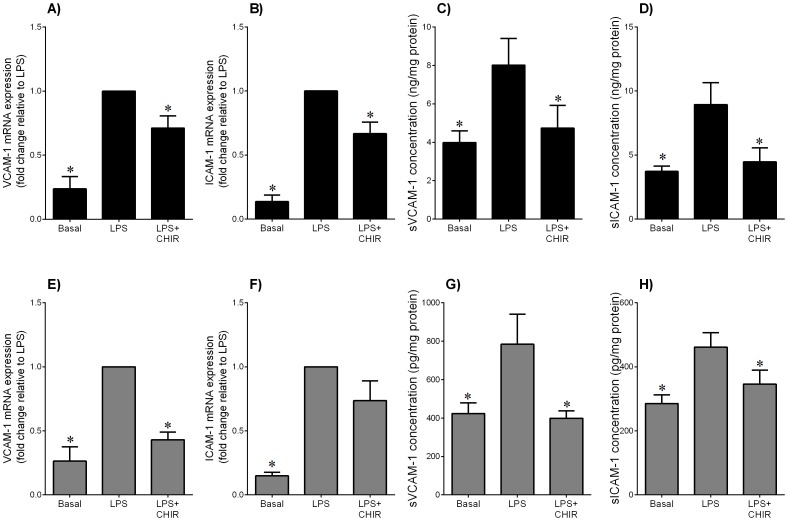
Effect of GSK3 inhibitor CHIR99021 on LPS-induced adhesion molecules in adipose tissue and skeletal muscle. Human (**A–D**) omental adipose tissue and (**E–H**) skeletal muscle were incubated with 10 µg/ml LPS in the absence or presence of 10 µM CHIR99021 (CHIR) for 20 h (n = 6 patients). (**A,B,E,F**) Gene expression for VCAM-1 and ICAM-1 was analysed by qRT-PCR. Gene expression was normalised to GAPDH mRNA expression and the fold change was calculated relative to LPS. Data displayed as mean ±SEM. *P<0.05 vs. LPS (one-way ANOVA). (**C,D,G,H**) The incubation medium was assayed for concentration of sVCAM-1 and sICAM-1 release by ELISA. Data displayed as mean ±SEM. *P<0.05 vs. LPS (one-way ANOVA).

**Figure 9:**
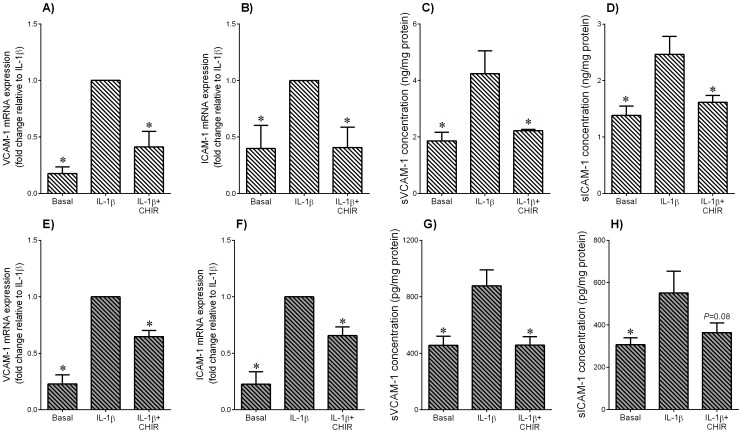
Effect of GSK3 inhibitor CHIR99021 on IL-1β-induced adhesion molecules in adipose tissue and skeletal muscle. Human (**A–D**) omental adipose tissue and (**E–H**) skeletal muscle were incubated with 1 ng/ml IL-1β in the absence or presence of 10 µM CHIR99021 (CHIR) for 20 h (n = 6 patients). (**A,B,E,F**) Gene expression for VCAM-1 and ICAM-1 was analysed by qRT-PCR. Gene expression was normalised to GAPDH mRNA expression and the fold change was calculated relative to IL-1β. Data displayed as mean ±SEM. *P<0.05 vs. IL-1β (one-way ANOVA). (**C,D,G,H**) The incubation medium was assayed for concentration of sVCAM-1 and sICAM-1 release by ELISA. Data displayed as mean ±SEM. *P<0.05 vs. IL-1β (one-way ANOVA).

Statistics was performed on the normalised data unless otherwise specified. All statistical analyses were undertaken using GraphPad Prism (GraphPad Software, La Jolla, CA, USA). For Figs. 1 and 2, an unpaired Student's t-test was used to assess statistical significance between normally distributed data; otherwise, the nonparametric Mann-Whitney U test was used. For Figs. 3–9, the homogeneity of data was assessed by the Bartlett test, and when significant, the data were logarithmically transformed before further analysis using a one-way ANOVA (using LSD correction to discriminate among the means). Statistical significance was ascribed to *P* value <0.05. Data were expressed as mean ± standard error of the mean (SEM).

## Results

### GSK3α/β activity is increased in adipose tissue and skeletal muscle from obese pregnant women and women with GDM

Adipose tissue and skeletal muscle was obtained from 12 women with NGT (6 non-obese and 6 obese), 12 BMI-matched women with GDM controlled by diet (6 non-obese and 6 obese). Demographic data of all participants involved in this study are summarised in Table 1 for adipose tissue and Table 2 for skeletal muscle. There was no difference in maternal pre-pregnancy BMI, maternal BMI at delivery, maternal age, gestational weight gain, fetal birthweight, or gestation age at delivery. Fasting, one hour and two hour plasma glucose during the antenatal OGTT were significantly higher in women with GDM when compared to NGT pregnant women.

Western blotting was used to determine the activity of GSK3α/β. The inactivation of GSK3 activity can be induced by phosphorylation at one of its N-terminal serine (Ser) residues: Ser21 for GSK3α and Ser9 for GSK3β. In adipose tissue, the expression of the phosphorylated GSKα (p-GSKα) isoform was very low and thus could not be analysed further by densitometry. For both adipose tissue and skeletal muscle, total GSK3β was used for normalisation of the phosphorylated GSK3 data. As shown in Fig. 1, p-GSK3β was lower in adipose tissue obtained from non-obese and obese women with GDM when compared to BMI-matched NGT controls. The data for the p-GSKα and p-GSKβ isoforms in skeletal muscle are presented in Fig. 2. There was no difference in the expression of p-GSKα between NGT and BMI-matched women (Fig. 2A,D). On the other hand, the expression of p-GSK3β was lower in skeletal muscle obtained from non-obese and obese women with GDM when compared to BMI-matched NGT controls (Fig. 2B,E). It should be noted that in the NGT patients, there was no difference in p-GSK3α/β protein expression between lean and obese subjects for both tissues (data not shown).

### The GSK3α/β inhibitor CHIR99021 decreases LPS- or IL-1β-induced pro-inflammatory cytokines in adipose tissue and skeletal muscle

The above studies show that GSK3β activity is increased in adipose tissue and skeletal muscle of women with GDM. Thus, the next aim was to determine the effect of the GSK3 inhibitor CHIR99021 on inflammation induced expression and secretion of pro-inflammatory cytokines. The effect of CHIR99021 on GSK3β protein expression is demonstrated in Fig. 3A. For these studies, inflammation was induced by the TLR4 ligand bacterial endotoxin LPS and the pro-inflammatory cytokine IL-1β. For LPS treatment, the data for adipose tissue and skeletal muscle are depicted in Figs. 3 and 4, respectively; the data for IL-1β for adipose tissue and skeletal muscle are shown in Fig. 5. Treatment with LPS induced a significant increase in TNF-α, IL-1β and IL-6 mRNA expression and secretion in both adipose tissue and skeletal muscle (Figs. 3 and 4, respectively). CHIR99021 significantly decreased LPS-induced TNF-α, IL-1β and IL-6 mRNA expression and secretion. Treatment with IL-1β increased IL-6 gene expression and release from both tissues and co-treatment with CHIR99021 significantly attenuated this increase in IL-6 expression and secretion (Fig. 5). The levels of TNF-α were unable to be detected in tissues treated with IL-1β. Of note, there was no effect of CHIR99021 on cytokine release under basal conditions (data not shown).

### The GSK3α/β inhibitor CHIR99021 decreases LPS- or IL-1β-induced pro-inflammatory chemokines in adipose tissue and skeletal muscle

IL-8 and MCP-1 are two pro-inflammatory chemokines that play an important role in the initiation and maintenance of the inflammatory response [32]. For both adipose tissue (Fig. 6A–D) and skeletal muscle (Fig. 6E–H), LPS induced a significant increase in IL-8 and MCP-1 mRNA expression and secretion. In the presence of CHIR99021, the increase in chemokine expression and release induced by LPS was significantly attenuated. The effect of CHIR99021 on chemokine expression in the presence of IL-1β is presented in Fig. 7A–D for adipose tissue and Fig. 7E–H for skeletal muscle. In adipose tissue, CHIR99021 significantly decreased IL-8 and MCP-1 mRNA expression and MCP-1 secretion (Fig. 7A,B,D). There was however, no effect of CHIR99021 on IL-1β induced IL-8 release in adipose tissue (Fig. 7C). In skeletal muscle, CHIR99021 significantly decreased IL-8 gene expression and release (Fig. 7E,G); there was no effect on MCP-1 gene expression and release (Fig. 7F,H). There was no effect of CHIR99021 on chemokine release under basal conditions (data not shown).

### The GSK3α/β inhibitor CHIR99021 decreases LPS- or IL-1β-induced expression of adhesion molecules in adipose tissue and skeletal muscle

VCAM-1 and ICAM-1 are two members of the immunoglobulin gene superfamily that are critical in the recruitment and infiltration of inflammatory cells; the soluble forms of these proteins are secreted from cells during inflammation and are increased in GDM pregnancies. The data for LPS are presented are presented in Fig. 8; the data for IL-1β in Fig. 9. Treatment of adipose tissue (Figs. 8A–D; 9A–D) or skeletal muscle (Figs. 8E–H; 9E–H) with LPS or IL-1β induced a significant increase in VCAM-1 and ICAM-1 mRNA expression. This was associated with an increased release of sVCAM-1 and sICAM-1. CHIR99021 significantly decreased the increase gene expression and secretion in both tissues except for LPS-induced ICAM-1 mRNA expression in skeletal muscle (Fig. 8F) and IL-1β-induced sICAM-1 release for skeletal muscle (Fig. 9H). There was no effect of CHIR99021 on adhesion molecules under basal conditions (data not shown).

### The GSK3α/β inhibitor SB216763 decreases LPS-induced pro-inflammatory cytokines and chemokines in adipose tissue

**Figure 10:**
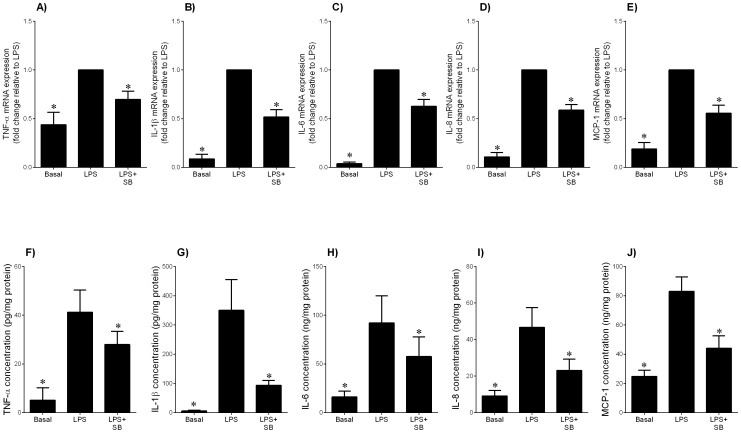
Effect of GSK3 inhibitor SB216763 on LPS-induced pro-inflammatory cytokines and chemokines in adipose tissue. Human omental adipose tissue was incubated with 10 µg/ml LPS in the absence or presence of 20 µM SB216763 (SB) for 20 h (n = 5 patients). (**A–E**) Gene expression for TNF-α, IL-1β, IL-6, IL-8 and MCP-1 was analysed by qRT-PCR. Gene expression was normalised to GAPDH mRNA expression and the fold change was calculated relative to LPS. Data displayed as mean ±SEM. *P<0.05 vs. LPS (one-way ANOVA). (**F–J**) The incubation medium was assayed for concentration of TNF-α, IL-1β, IL-6, IL-8 and MCP-1 release by ELISA. Data displayed as mean ±SEM. *P<0.05 vs. LPS (one-way ANOVA).

CHIR99021 which is the most selective inhibitor of GSK3α/β reported to date [30]. In order to confirm a role for GSK3 in inflammation, additional experiments were performed in adipose tissue using another GSK3 inhibitor SB216763. SB216763 is a potent and selective, ATP-competitive GSK3 inhibitor that is equally effective at inhibiting human GSK3α and GSK3β. The effect of SB216763 on LPS-induced inflammation is presented in Fig. 10. SB216763 significantly decreased LPS-induced mRNA expression and secretion of pro-inflammatory cytokines (TNF-α, IL-1β and IL-6) and chemokines (IL-8 and MCP-1).

## Discussion

The novel findings of this study are that GDM is associated with increased GSK3 activity in omental adipose tissue and skeletal muscle. Chemical inhibition of GSK3 with the synthetic compound CHIR99021 could efficiently prevent the expression and release of pro-inflammatory mediators in omental adipose tissue and skeletal muscle that were stimulated with TLR4 ligand and bacterial product LPS and the pro-inflammatory cytokine IL-1β. Collectively, these results show the importance of GSK in regulating inflammation in pregnant adipose tissue and skeletal muscle.

GSK3 activity is regulated by a number of mechanisms. The most well-defined regulatory mechanism is, however, inhibition of the activity of GSK3 by phosphorylation of a regulatory serine in either of the two isoforms of GSK3, ser9 in GSK3β or ser21 in GSK3α [33]. In this study, serine phosphorylated GSK3β expression was lower in adipose tissue and skeletal muscle obtained from women with GDM; thus, suggesting increased GSK3β activity in pregnant GDM tissues. These findings are in concordance with studies in non-pregnant women. Increased expression of GSK3 has been reported in skeletal muscle from type 2 diabetes patients [29] and in adipose tissue of diabetes-prone C57/BL6 mice [25]. In mammals, GSK3 exists as two isoforms: GSK3α and GSK3β which are structurally similar but encoded by distinct genes and have molecular weights of 51 and 47 kD, respectively [33]. Although the two isoforms of GSK have similar functions, they are not functionally redundant as deletion of GSK3β leads to embryonic lethality at E16 that cannot be rescued by GSKα [34].

A strength of this study is the well-defined study population that was used for assessment of GSK3 expression. Patients were excluded for maternal or clinical factors which may influence the maternal inflammatory profile such as asthma, preeclampsia, pre-existing diabetes, hypertension and smoking. In addition, tissues were obtained from women at the time of term Caesarean section (non-labouring). Importantly, patients were also matched for gestational weight gain and maternal BMI. A further strength of this study was the inclusion of tissues from GDM women controlled by diet therapy only ensuring that the changes in GSK3β expression are not due to insulin therapy. A limitation of this study is that maternal plasma was not available from the GDM patients to assess the concentrations of pro-inflammatory cytokines. Nevertheless, previous studies have demonstrated increased circulating levels of TNF-α [39], MCP-1 [35], IL-1β [36] and IL-6 [37] in patients with GDM.

Excessive release of pro-inflammatory molecules from adipose tissue and skeletal muscle may be involved in the pathogenesis and/or progression of GDM. It is now well-established that pregnant adipose tissue expresses and secretes a variety of pro-inflammatory cytokines and chemokines [12]–[18], [38]–[40]. Notably, their expression and production is enhanced in obese pregnant women and/or women with GDM [16]–[19]. However, pro-inflammatory cytokines are also secreted by other insulin target tissues including skeletal muscle [13], [15], [40]. In addition to pro-inflammatory cytokines, recent studies support a role for bacterial infection in the development of diabetes [23], [41], [42]. Of note, women with periodontal disease have increased prevalence of GDM [43], [44].

Sterile inflammation or bacterial infections may induce maternal or placental inflammation associated with GDM pregnancies. In support, TNF-α induces pro-inflammatory cytokine expression in human placenta, and adipose tissue and skeletal muscle from pregnant women [13]. Bacteria or bacterial products (e.g., LPS) from the inflamed or infected site may enter the circulation and trigger a maternal systemic inflammatory response. Indeed, *in vitro*, bacterial endotoxin LPS induces the expression of pro-inflammatory cytokines in adipose tissue and skeletal muscle from pregnant women [13], [15]. Further support for the role of inflammation and infection in inducing inflammation comes from the findings of this study. That is, sterile inflammation (i.e. IL-1β) or bacterial infection (i.e. LPS) induced the expression and secretion of pro-inflammatory cytokines from human pregnant adipose tissue and skeletal muscle.

Given the central role of pro-inflammatory cytokines and infection in regulating the inflammatory response, studies were undertaken to determine if GSK3 plays a role in this process. In this study, the GSK3 inhibitor CHIR99021 decreased the expression and secretion of the pro-inflammatory cytokine TNF-α, IL-1β and/or IL-6 and the chemokines IL-8 and MCP-1 in adipose tissue and skeletal muscle stimulated with LPS or IL-1β. To my knowledge, this is the first study to report GSK3 regulates inflammation in skeletal muscle and adipose tissue. However, these findings are in agreement with previous studies in various cells and tissues. GSK3 activity is necessary for TLR-induced production of the pro-inflammatory cytokines IL-6, IL-1β, MCP-1 and TNF-α in monocytes and peripheral blood mononuclear cells [24]. Additionally, GSK3 can regulate also inflammation in the central nervous system, airway smooth muscle cells, adipocytes and dendritic cells [45]–[47]. Notably, several animal models of inflammation have demonstrated an important role for GSK3β in modulating stimulus-induced production of several cytokines and the subsequent development of disease symptoms. For example, inhibition or deletion of GSK3β was protective against experimental peritonitis and arthritis [48], renal dysfunction and hepatotoxicity associated with endotoxemia [49], and endotoxin shock [24].

The cell adhesion molecules VCAM-1 and ICAM-1 are two members of the immunoglobulin gene superfamily that are critical in the recruitment and infiltration of inflammatory cells to sites of injury. ICAM and VCAM, which are constitutively expressed in a few cell types, are induced by a various pro-inflammatory stimuli, including IL-1β and LPS [50]. The soluble forms of ICAM-1 and VCAM-1 are detected in the systemic circulation due to the proteolytic cleavage at the cell surface. Levels of sICAM-1 and sVCAM-1 are higher with insulin resistance, obesity and type 2 diabetes [51]. Similarly, women with GDM have increased circulating sICAM-1 and sVCAM-1 levels [52]. My recent studies have also shown that the expression and secretion of markers of endothelial cell dysfunction are increased in adipose tissue from women with GDM [17]. In this study, treatment with LPS or IL-1β increased the expression and secretion of VCAM-1 an ICAM-1 from adipose tissue and skeletal muscle. This increase was significantly decrease by pre-treatment with the GSK3 inhibitor CHIR99061. To my knowledge, this is the first study to report that GSK3 regulates adhesion molecules in adipose tissue and skeletal muscle. However, inhibition of GSK3β decreased TNF-α induced expression of ICAM-1 and VCAM-1 in brain endothelial cells [53].

Pro-inflammatory cytokines or bacterial infections may also induce insulin resistance associated with GDM pregnancies [5]–[7]. In support, TNF-α is a significant predictor of insulin resistance during pregnancy [54]; IL-1β interferes with the insulin signalling pathway in adipose tissue from pregnant women [12] and human placenta [55]; and IL-6 stimulates trophoblast fatty acid accumulation [56] and increases trophoblast System A amino acid transporter activity and expression [57]. Bacterial infections have also been shown to induce insulin resistance in a number of *in vitro* and *in vivo* models [58]–[60]. Collectively, these findings suggest that sterile inflammation or bacterial infections, by increasing peripheral insulin resistance and/or placental nutrient transport, may contribute to the increased fat deposition observed in infants of women with GDM [61]. Future studies to determine the role of GSK3 in regulating the insulin signalling pathway in adipose tissue and skeletal muscle are warranted.

The mechanism by which GSK3 exerts its inflammatory actions in pregnant adipose tissue and skeletal muscle is not known. However, GSK3 has been shown to differentially activate a number of transcription factors such as AP-1, cAMP-response element binding protein (CREB), signal-transducer and activator of transcription 1-3 (STAT1-3), transcription factor 7-like 2 (TCF712), β-catenin, and nuclear factor-κB (NF-κB). With respect to the regulation of inflammation by GSK3, NF-κB is the most widely studied mechanism [24], [62]. It has recently been shown that β-catenin may play an important role in GSK-mediated regulation of NF-κB gene transcription. Specifically, inhibition of GSK3 leads to the translocation of β-catenin from the cytoplasm to the nucleus, which in turn can block the activity of the pro-inflammatory transcription factor NF-κB [63]. Whether GSK3 regulates LPS- or IL-1β- induced pro-inflammatory cytokines and adhesion molecules in adipose tissue and skeletal muscle via β-catenin and/or NF-κB, is not known and warrants further investigation. It should, however, be noted that my previous studies have shown that NF-κB regulates the secretion of pro-inflammatory cytokines in pregnant adipose tissue and skeletal muscle [38], [40]. Furthermore, there are numerous studies demonstrating the importance of NF-κB in insulin resistance and type 2 diabetes [64], [65].

The secretion of pro-inflammatory cytokines and adhesion molecules is higher in women with GDM [17], [38]. A limitation of this study was that the effect of the GSK3 inhibitors on the release of pro-inflammatory cytokines and adhesion molecules was not assessed in adipose tissue and skeletal muscle obtained from women with GDM. In this study, LPS and IL-1β were used to mimic a GDM environment. Thus, the findings of this current study must be interpreted with some caution. A further limitation of this study is that the GSK3 isoform responsible for regulating the expression and secretion of pro-inflammatory cytokines and adhesion molecules in the presence of LPS or IL-1β is not known. Knockout or overexpression studies of each isoform would be useful in elucidating the exact role of GSK3α and GSK3β in regulating inflammation.

In conclusion, the data presented in this study show that GSK3 activity is increased in omental adipose tissue and skeletal muscle obtained from women with GDM. Induction of the gene expression and secretion of pro-inflammatory cytokines and chemokines, and adhesion molecules in the presence of LPS or IL-1β were significantly decreased by the GSK3 inhibitor CHIR99021. These results indicate that GSK3 may play an important role in inflammation that is evident in GDM pregnancies [12], [38], [66]–[68]. Inflammation play a central role in mediating insulin resistance and thus may contribute to the fetal overgrowth and or increased fat deposition observed in infants of women with GDM [61]. It would be of interest to determine the effect of the GSK3 inhibitor CHIR99021 on both maternal and fetal metabolic profiles and outcomes in an animal model of GDM. Indeed, a single oral dose (30 mg/kg) of CHIR99021 has been shown to enhance *in vivo* glucose metabolism in rodent models of type 2 diabetes [30].
